# Impact of Subthalamic Deep Brain Stimulation on Hyposmia in Patients With Parkinson's Disease Is Influenced by Constipation and Dysbiosis of Microbiota

**DOI:** 10.3389/fneur.2021.653833

**Published:** 2021-04-06

**Authors:** Chao Li, Ying Hou, Xu Wang, Yue-xuan Li, Feng Li, Chao Zhang, Wei-guo Li

**Affiliations:** ^1^Department of Neurosurgery, Qilu Hospital of Shandong University, Jinan, China; ^2^Department of Brain Function Remodeling, Institute of Brain and Brain-Inspired Science Research, Shandong University, Jinan, China

**Keywords:** gut microbiota, DBS, hyposmia, PD, olfactory dysfunction, constipation

## Abstract

**Background:** Non-motor symptoms in PD usually arise at very early stage and vary during the whole disease progression. Deep brain stimulation (DBS) is considered as a highly efficient treatment option for PD's motor function. However, the effect of DBS on NMS, especially hyposmia, has not been fully understood and the deep connection between different NMS such as hyposmia and constipation is still unknown.

**Objective:** The objective of this study was to evaluate the therapeutic effect of DBS on hyposmia in PD patients with or without constipation and find potential factors which might influence the efficacy.

**Methods:** A retrospective analysis of 65 PD patients accepted STN-DBS operation in Qilu Hospital during 2019–2020 were conducted to evaluate the exact therapeutic effect of DBS on hyposmia in PD. Sub-group analyses about the relationship between hyposmia and constipation were carried out. Analysis of flora in nasal mucosa was also conducted to evaluate the abundance and variety in different PD groups.

**Results:** Our study showed that DBS had clearly improved olfactory function in Parkinson patients (*P* = 0.012) and subgroup analysis found that PD patients with constipation have lower olfactory function scores (25.27 ± 3.44 vs. 33.90 ± 6.633, *p* = 0.014) and worse improvement after DBS operation (ΔTDI 12.11 ± 3.2 vs. 8.78 ± 2.91, *p* = 0.0072). Analysis of flora indicated the obvious discrepancy on olfactory function scores and degree of improvement might be related to the abundance and dysbiosis of microbiota.

**Conclusion:** In summary, this article presents a study on PD with hyposmia and constipation after DBS operation, explored the relationship between different NMS and offer a potential explanation on why PD patients with constipation usually have worse olfactory function for the less abundance and variety of microbiota.

## Introduction

Parkinson's disease (PD) is the second most common neurodegenerative disease (1). By 2030, morbidity is expected to double in developing countries (2). A major challenge for patients is maintaining a satisfactory quality of life with non-motor symptoms in the progression of PD. Traditional drugs, such as levodopa, have been reported to bring little benefits for, or to sometimes even worsen, non-motor symptoms in PD (3).

Non-motor symptoms (NMS), such as sleep disturbances, constipation, cognition, olfactory dysfunction, and changes in mood in Parkinson's disease (PD) usually have a high incidence and emerge from a very early phase (4). A total of 90% of PD patients suffer hyposmia several years before the onset of motor symptoms (5). Rapidly increasing data have shown that olfactory parameters [(odor threshold (OT), odor discrimination (ODI), and odor identification (OI)] are gradually impaired along with the neurodegenerative progression of PD (6). However, levodopa seemed to have no effect on improving hyposmia in patients with PD (7). Thus, studies have shed light on other interventions, such as deep brain stimulation (DBS), for amelioration of NMS other than motor function benefits.

A review of literature reveals that the effect of DBS on hyposmia in PD has not been fully understood, and contradictory data have been reported by different research teams. For instance, some studies indicated that DBS in the STN did not change the olfactory function score in the acute post-operative process within 1 month of observation (8), while others have shown that DBS treatment provides an obvious improvement in OD in long-term follow-up (3–12 months) (9–11). All the sample sizes of the reviewed studies are limited, and factors that may influence the therapeutic effect of DBS on hyposmia are unclear.

With further understanding of the mechanisms of PD, dysbiosis of microbiota is considered to be associated with pathophysiological changes not only in the gastrointestinal system but also in other enteric and central nervous systems. As the nasal mucosa and intestine are the first barriers to protect the body from pathogenic bacteria, they are the most affected by dysbiosis of microbiota. Thus, we believed that there was a potential relationship between idiopathic constipation and hyposmia in PD, and this hypothesis has not been reported thus far (12). Moreover, the influence of microbiota on hyposmia and constipation in PD, especially during DBS surgery, has been rarely discussed, and further studies are needed.

## Methods and Materials

### Patient Selection

This study was performed at the Qilu Hospital of Shandong University, Movement Disorders Clinic of Functional Neurosurgery, between March 2019 and September 2020. The ethics committee of the hospitals approved the study protocol (protocol number: KYLL-202008-065), and the entire study process was performed according to the Declaration of Helsinki. All patients provided informed consent before participating in the study.

A total of 65 patients with idiopathic PD and 50 healthy controls were recruited for the study. The criteria of the United Kingdom Parkinson's Disease Society Brain Bank (UPDRS)were used to diagnose idiopathic PD. Controls were recruited from partners and acquaintances of healthy people without stroke, idiopathic trauma, multiple sclerosis, progressive supranuclear palsy, and a history of drugs that might lead to PD syndrome. All patients underwent bilateral subthalamic deep brain stimulation (STN-DBS) surgery.

Enrolled patients underwent olfactory tests 1 h after levodopa intake to assess odor function. The same olfactory tests were performed in the control group. After the test, 35 of 65 patients were diagnosed with hyposmia (54.7%). DBS stimulus parameters were as follows: amplitude, 1.5–3.4 V; pulse width was 60 ms; and frequency, 130–180 Hz. After the patients were discharged from the hospital, olfactory tests were repeated in the post-operative period (at 3rd month) with both stimulation and drug “on.”

### Olfactory Tests

The Sniffin-Sticks Test (Burghardt, Wedel, Germany) with Chinese validation was used for olfactory evaluation. Olfactory function was evaluated using three parameters: tests for odor threshold (OT), odor discrimination (ODI), and odor identification (OI). The test was carried out in an odorless, well-ventilated room. The tester (Ms. Hou and Mr. Li) wore odorless gloves. Each pencil was moved 2 cm close to both nostrils once for 3 s. The subjects were told to smell it. A 3 min break was taken between the three parts of the test by the sequence of threshold-discrimination-identification, according to Saatçi et al. (13).

### Constipation Score System

We evaluated the severity of constipation in PD according to the Constipation Scoring System (CSS). The questionnaire included different sections, and each section score ranged from 0 (normal) to 4 (severe) except for defecation (ranging from 0 to 2). Each individual score was added to obtain a global score. According to the global score, constipation can be classified as mild (score 1–5), moderate (6–10), severe (11–15), or very severe (15–30). We named the mild and moderate groups as constipation-negative group and the severe as well as very severe groups as constipation-positive group for further study.

### 16S Real-Time Quantitative PCR and 16S Data Analysis

Bacterial DNA were extracted from samples obtained from the nasal mucosa of patients with PD by throat swab. Frozen tissue was extracted using a Thermo Fisher MagMAX Pathogen DNA Isolation kit according to the manufacturer's instructions. 16S real-time quantitative polymerase chain reaction (PCR) was conducted using universal bacterial primers for the V6 region of the 16S ribosomal RNA (rRNA) region: 5′-CNACGCGAAGAACCTTANC-3′, 5′-ATACGCGARGAACCTTACC-3′, 5′-CTAACCGANGAACCTYACC-3′, 5′-CAACGCGMARAACCTTACC-3′, and 5′-CGACRRCCATGCANCACCT-3′. The obtained data were analyzed, and relative abundances were converted to read counts by multiplying the number of reads. Sample reads were normalized based on a related sequencing library by a factor representing the ratio of the average number of reads/sample in the specific library to the overall average across libraries. Samples with fewer than 1,000 normalized reads (including negative controls) and species with relative abundances of <10^−4^ were excluded from further analysis.

### Heat Map of Bacterial Flora Distribution

Excel 2016 with a Power-map plug-in was used to analyze the distribution of bacterial flora. Information on PD patients' present addresses was imported into the power map as well as the corresponding percentage of target microbiota (Stenotrophomonas, Lactobacillus, Neisseria, and Veillonella). Subsequently, a heat map was generated and exported.

### Statistical Analysis

Prism software (version 8.01) was used for statistical analysis. Descriptive values are reported as mean ± standard deviation. Unpaired *t*-tests were used to estimate the potential relationship between DBS and clinical pathological outcomes, and a paired *t*-test was applied to evaluate the effect of DBS surgery on SST and CSS scores. Spearman correlation was used to assess the relationship between the change in constipation scores and changes in olfactory scores. ANOVA was used to compare the therapeutic effects of DBS on olfactory function in different cities. Statistical significance was accepted when the two-sided *p* < 0.05.

## Results

### Participant Characteristics

A total of 65 PD patients who underwent STN-DBS surgery and 50 healthy individuals were included in this study. Table 1 summarizes the baseline characteristics of the enrolled patients. The two groups were well-balanced for age, sex, education, and address location. The median age of the participants was 60.02 years in the normal group and 58.8 in PD patients with no obvious difference (*P* > 0.05). We also found no obvious differences in gender, education, and address location (*P* > 0.05). The UPDRS III score significantly improved after the patients underwent DBS. The mean pre-operation UPDRS III score was 33.46, the mean of post-UPDRS III on stimulation was 13.05, and the post-UPDRS III off stimulation was 31.21% (33.46 ± 20.2 vs. 13.05 ± 10.2, *p* < 0.001; 31.21 ± 18.3, vs. 13.05 ± 10.2, *p* < 0.001), indicating that the DBS was working fine during the observation. Comparing the two groups in this study, we found that the patients with PD had a significantly higher ratio of olfactory dysfunction (*n* = 2 vs. 35, 4 vs. 54.7%) and constipation (*n* = 11 vs. 39, 22 vs. 60.9%) (Table 1), which is in accordance with results from other studies (14). Among them, we found that there were 28 persons presenting with both hyposmia and constipation in the group.

**Table 1:** Participant characteristics.

**Total number**	**Normal**	**PD**	***P*-value**
Age, y, median (IQR) [range]	60.02	58.8	NS
Sex, *n* (%)	24 (48)	30 (46.9)	NS
Disease duration, y, median (IQR) [range]	NA	11.91	NA
Levodopa dose equivalent, mg, median (IQR) [range]	NA	180.2	NA
Education, college degree	9.9	9.3	NS
Current address (costal city), *n* (%)	16 (33.3)	21 (32.2)	NS
Pre-op UPDRSIII score	NA	33.5	NA
Post-op UPDRSIII score (on stimulation)	NA	13	NA
Post-op UPDRSIII score (off stimulation)	NA	31.2	NA
MMSE	28.1	20.4	0.011
Diplopia, *n* (%)	3 (5)	48 (75.2)	<0.001
Pollakisurie, *n* (%)	8 (16)	34 (53.1)	<0.001
Hyposmia, *n* (%)	2 (4)	35 (54.7)	<0.001
Constipation, *n* (%)	11 (22)	39 (60.9)	<0.001

### PD Patients Showed Improved Olfactory Function Scores After STN-DBS Surgery

**Figure 1:**
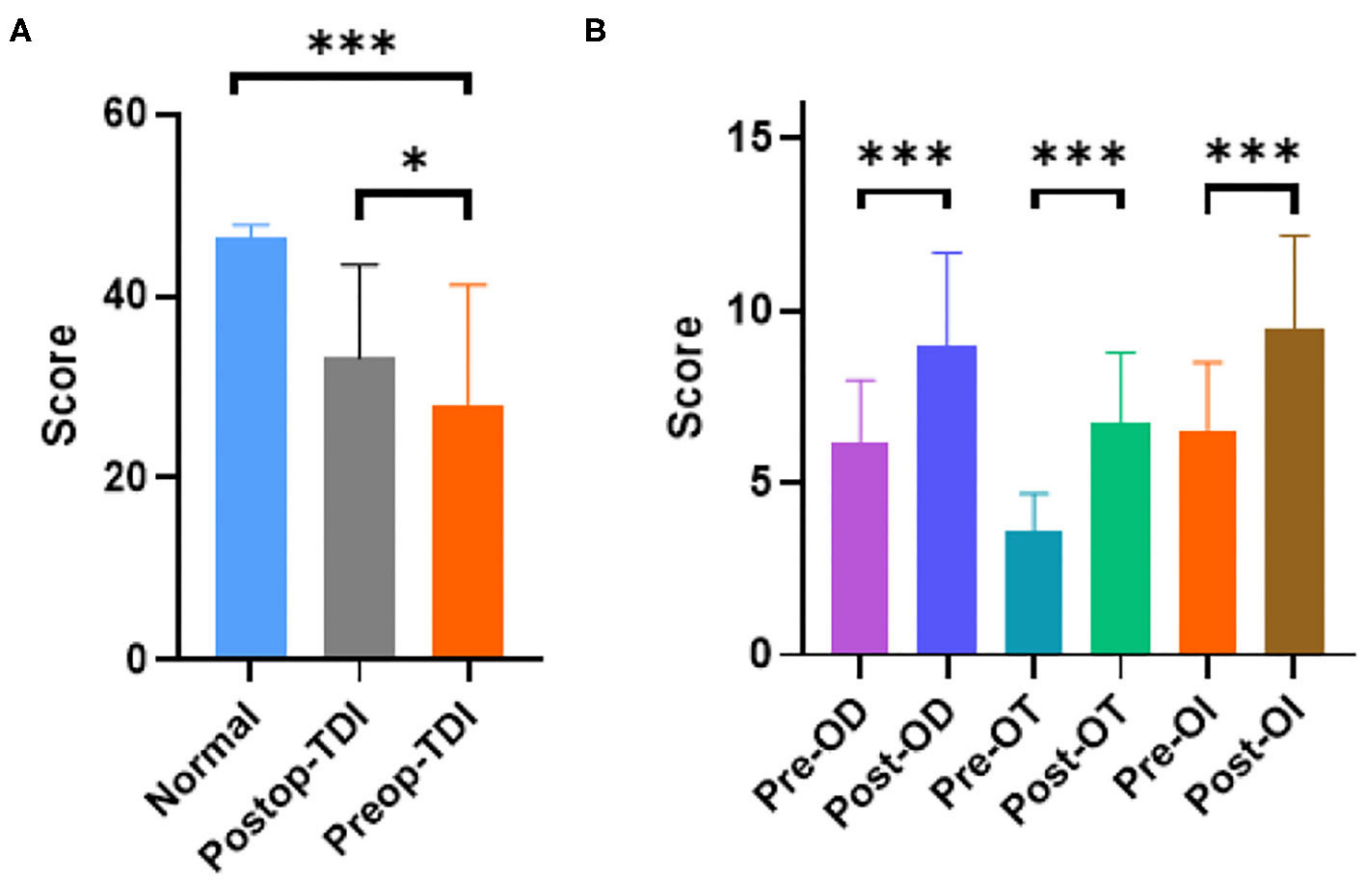
PD patients have obvious smell disorders compared with the normal population, and DBS can improve the smell disorders in patients. **(A)** PD group had obvious lower TDI score (*P* < 0.01 unpaired student's *t*-test. Error bar = mean ± standard deviation) and DBS surgery could improve it among PD patients (*P* = 0.012 paired student's *t*-test. Error bar = mean ± standard deviation). **(B)** Different parameters (OD, OT, and OI) got improved by DBS (*P* < 0.01 paired student's *t*-test. Error bar = mean ± standard deviation). *p < 0.05 and ***p < 0.001.

The Sniffin-Sticks Test with Chinese validation was used for olfactory evaluation. Olfactory function was further evaluated using three parameters: tests for OT, ODI, OI, and a composite score of these three parts (TDI). Among these parts, there were obvious differences between the normal group and pre-operation group in percentage and olfactory score, shown in Table 1 (*n* = 0 vs. 35, 0 vs. 54.7%, *p* < 0.01) and Figure 1A (Normal vs. Pre-op: 46.40 ± 2.2 vs. 27.92 ± 13.3, *P* < 0.01). After DBS surgery, PD patients showed improved olfactory function scores between the pre- and post-operation groups (post-operative vs. pre-operative: 33.23 ± 10.25 vs. 27.92 ± 13.3, *P* = 0.012; Figure 1A). With further division of the scores into 3 different parts: ODI, OT, and OI, DBS showed more obviously positive effect on hyposmia patients, as seen in Figure 1B and Table 2 (Post-op vs. Pre-op: ODI, 8.97 ± 2.3 vs. 6.14 ± 1.55, *P* < 0.01; OT, 6.74 ± 2.41 vs. 3.60 ± 2.14, *P* < 0.01; 9.45 ± 2.52 vs. 6.45 ± 2.13, *P* < 0.01).

**Table 2:** DBS improved olfactory function scores with different parameters.

	**Normal**	**Pre-op**	**Post-op**	***P***
OT	15 ± 0.5	3.60 ± 5.2	6.74 ± 4.0	*P* < 0.001
ODI	15 ± 0.8	6.14 ± 4.2	8.97 ± 3.3	*P* < 0.001
OI	15 ± 0.6	6.45 ± 4.1	9.45 ± 3.2	*P* < 0.001
TDI	46.40 ± 2.2	27.92 ± 13.3	33.23 ± 10.25	*P* < 0.001

### PD Patients With Constipation Have Higher Ratio of Hyposmia, and Benefit Less on Olfactory Score After STN-DBS Surgery

**Figure 2:**
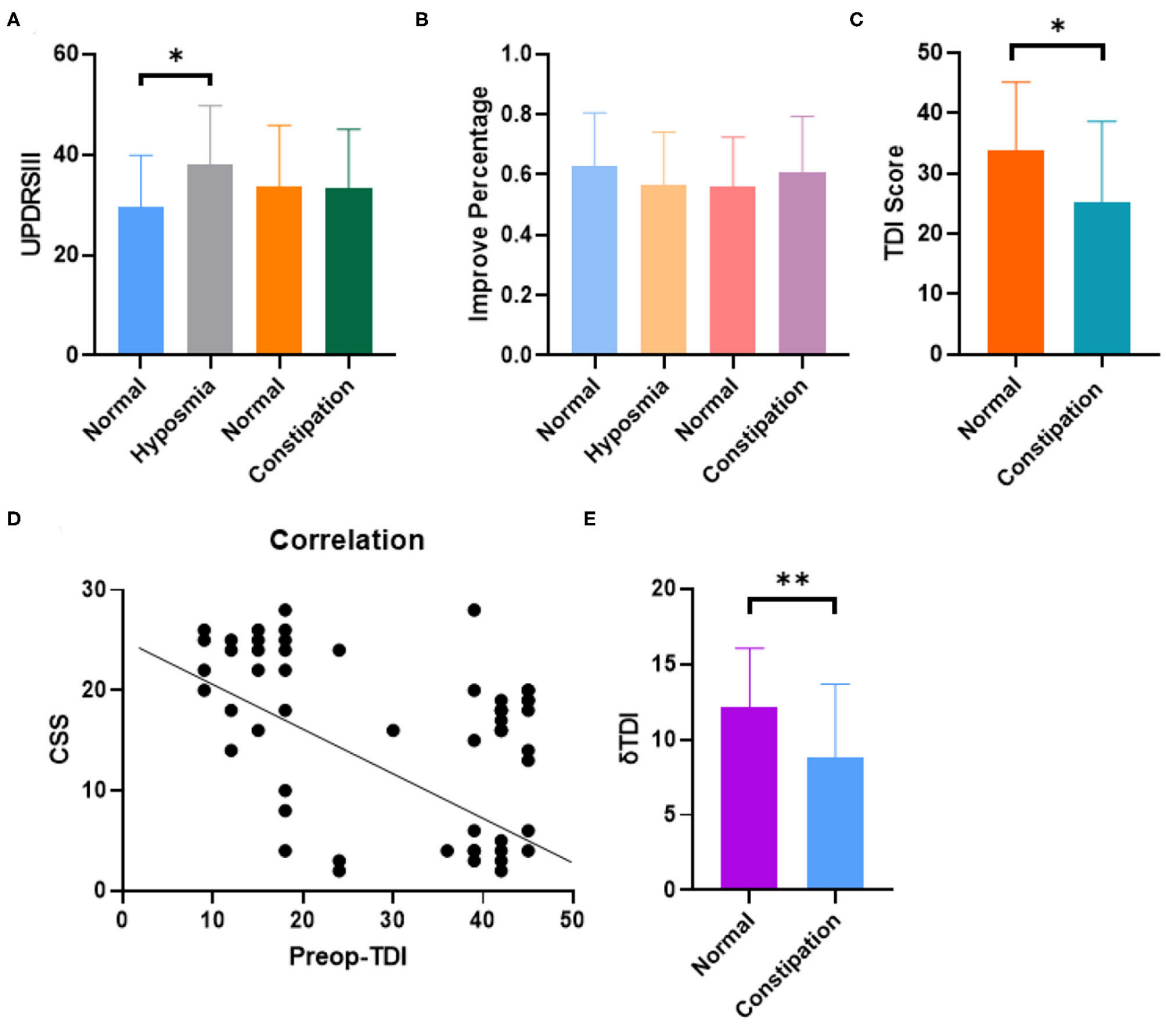
The rate of oldness disorder in PD patients with constipation was significantly increased, and the two scores were correlated to a certain extent, while the improvement of oldness score after DBS in patients with constipation was not ideal. **(A)** Hyposmia group showed higher UPDRSIII score and no obvious difference was observed in constipation and non-constipation group (*P* = 0.0381 Unpaired student's *t*-test. Error bar = mean ± standard deviation). **(B)** No obvious difference was observed in DBS improvement percentage within different groups (Error bar = mean ± standard deviation). **(C)** The olfactory function score is lower in constipation group than control group (*P* = 0.0145 Unpaired student's *t*-test. Error bar = mean ± standard deviation). **(D)** Constipation and hyposmia had negative correlation with each other (spearman, *r* = −0.4776, *p* < 0.01). **(E)** Constipation group had lower δTDI score indicating that PD patients with constipation got less therapeutic effect on hyposmia from DBS (*P* = 0.0072 Unpaired student's *t*-test. Error bar = mean ± standard deviation). *p < 0.05 and **p < 0.01.

We divided the patients into two groups: constipation and non-constipation. Interestingly, we found that the UPDRSIII score of the hyposmia group was slightly higher than that of the non-hyposmia group (38.00 ± 5.56 vs. 29.57 ± 4.98, *p* = 0.031, *p* < 0.05; Figure 2A), but with no obvious difference in DBS improvement percentage (0.62 ± 0.06 vs. 0.56 ± 0.04, *p* = 0.1584, *p* > 0.05; Figure 2B), which is in agreement with the results of previous studies (15, 16).

Interestingly, the constipation group did not show any difference compared to the non-constipation group with regard to the UPDRSIII score and DBS improvement percentage (Figures 2A,B). However, the ratio of hyposmia in the constipation group was much higher than that in the non-constipation group (71.8%, percentage = 28/39 vs. 26.9%, percentage = 7/26, *P* < 0.01). The TDI score reflecting olfactory function was also lower in the constipation group than in the non-constipation group (25.27 ± 3.44, 33.90 ± 6.633, unpaired *t*-test, *p* = 0.0145), as shown in Figure 2C. We then introduced CSS score to quantitatively analyze the level of constipation in PD patients. The results showed that there were clear correlations between the TDI score and CSS score (Spearman, *r* = −0.4776, *p* < 0.01; Figure 2D). In addition, we also found that constipation affected the therapeutic effect of DBS on hyposmia by calculating ΔTDI (ΔTDI = Post-TDI minus Pre-TDI) in different groups. In our study, ΔTDI was lower in the constipation group than in the non-constipation group (Normal PD without constipation vs. PD with constipation, ΔTDI = 12.11 ± 3.2 vs. 8.78 ± 2.91, *p* = 0.0072) Figure 2E and Table 3. Moreover, our study showed that DBS seemed to have no effect on improving constipation in patients with PD (Table 3). Summarizing all the results, we believe that if one PD patient has constipation, he would suffer a higher risk for hyposmia and benefit less from STN-DBS with regard to improvement of odor scores.

**Table 3:** DBS had different therapeutic effects on hyposmia between constipation and normal group.

	**PD_Normal**	**PD_Constipation**	***P***
Pre-op_TDI score	33.90 ± 8.3	25.27 ± 3.4	0.0145
Post-op_TDI score	38.10 ± 7.0	31.07 ± 2.6	0.095
Pre_CSS score	5.80 ± 1.0	21.51 ± 15.70	<0.001
Post_CSS score	5.30 ± 0.81	17.87 ± 12.57	<0.001

### The Microbial Population of PD Patients With Olfactory Disorder Was Significantly Different From That of the Normal Population

**Figure 3:**
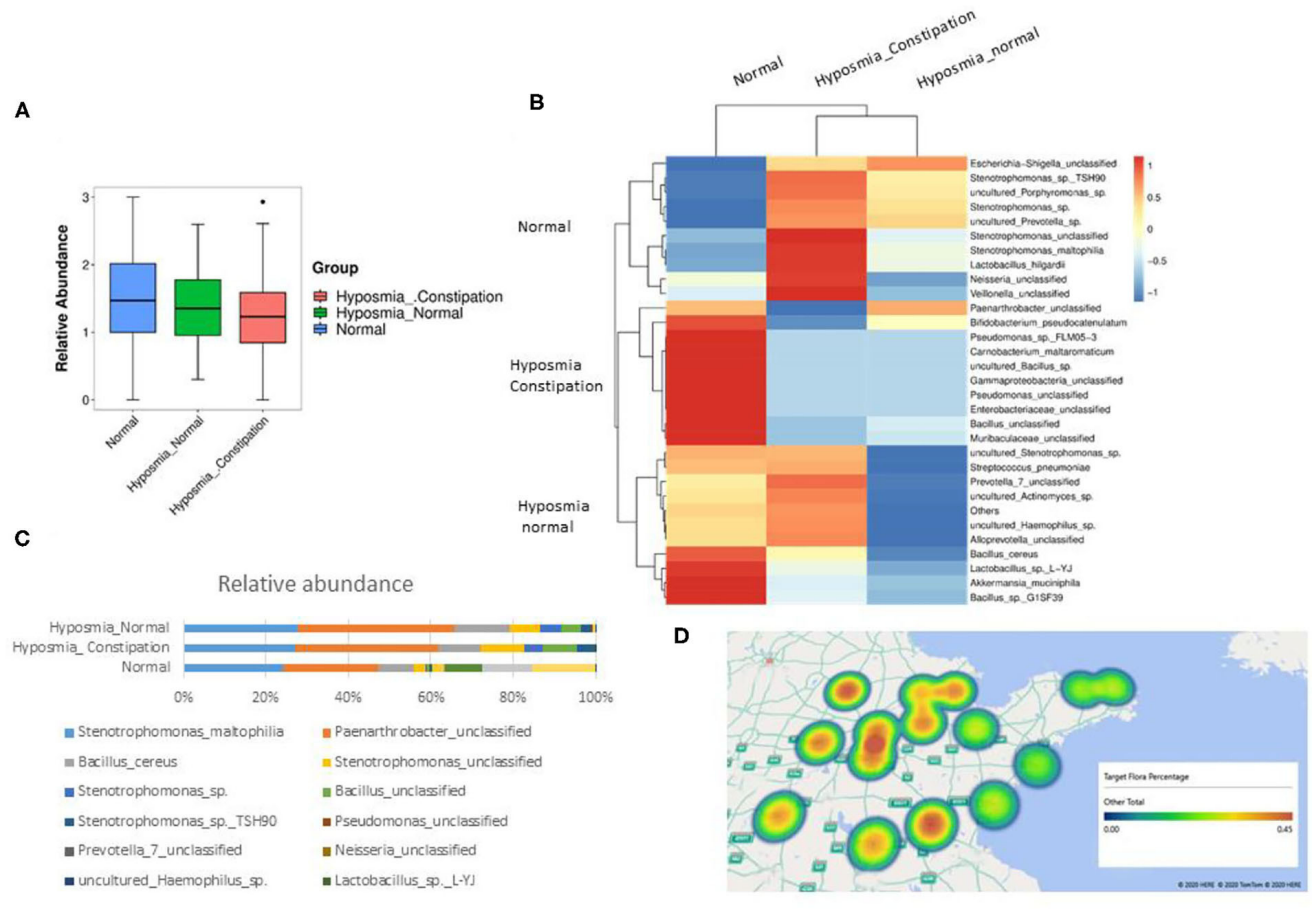
Significant difference was found between the flora of microbiota in PD patients with olfactory disturbance and normal population. **(A)** The bacteria abundance in PD patients with hyposmia is less than normal group, especially in patients with constipation and hyposmia (*P* = 0.038, *P* < 0.05, ANNNOVA, Error bar = mean ± standard deviation). **(B)** The variety of flora in hyposmia group is also less than control group. **(C)** The percentage of stenotrophomonas, lactobacillus, Neisseria, and veillonella are richer in hyposmia with constipation group. **(D)** The constituent of flora is related to the cities where PD patients live. Percentages of above microbiota (stenotrophomonas, lactobacillus, Neisseria, and veillonella) are higher in cities far away from sea.

To explore the potential mechanism of why constipation is usually accompanied by hyposmia in PD, we extracted bacterial DNA from the nasal mucosa and quantified a real-time quantitative PCR (qPCR) assay with universal primers 967F and 1064R specific for the bacterial ribosomal 16S gene [16S rDNA (ribosomal DNA)]. As shown in Figure 3A, the bacterial abundance in the nasal mucosa of PD patients with hyposmia was lower than that in the normal group, especially in patients with constipation and hyposmia (unpaired *t*-test, *P* = 0.038). Further analysis showed that the variety of flora in the hyposmia group was also less than that in the control group (Figure 3B). When hyposmia with or without constipation was compared, we found that the percentages of Stenotrophomonas, Lactobacillus, Neisseria, and Veillonella were greater in the hyposmia with constipation group (Figure 3C), which was also reported to be increased in the oral microbiota of PD patients compared with controls in another study (17). This may be the reason for the differences between the constipation and non-constipation groups. In addition, we also found that the constituents of flora are related to the cities where PD patients live. Percentages of the above microbiota (Stenotrophomonas, Lactobacillus, Neisseria, and Veillonella) are different in some specific places that are usually far away from the sea (Figure 3D). However, people from different places had a similar therapeutic effect on olfactory function of DBS, with no obvious difference (ANOVA test, *P* > 0.05).

## Discussion

Based on the common findings, our study confirmed that DBS could improve olfactory function in a larger number of patients. Herein, we also found that PD patients with constipation have a higher risk of developing hyposmia and obtain little benefits with DBS operation. Dysbiosis of microbiota may also play a key role in this phenomenon with a 16S examination of carefully gathered samples in PD nasal mucosa. To our knowledge, there is no report regarding this area, and our study provides further evidence for the origin of PD in the peripheral nervous system.

Nearly 90% of PD patients suffer hyposmia several years before the onset of motor symptoms, and levodopa showed no effect on reversing the process (14).

According to the hypothesis by Braak et al., the olfactory bulb was identified as the sparkle site at which the neurodegenerative process in PD starts and then spreads to the brainstem and cortical regions of the brain (7). Studies have found that the α-synuclein that appears in the olfactory bulb has the capacity to anterograde from the olfactory system into the temporal lobe along with the smell tract structure (18). Thus, hyposmia in PD was once thought to originate from the olfactory bulb and could not be modified by DBS.

However, some new findings (11, 13) together with our study, confirmed that DBS improved olfactory scores.

In these studies, DBS could obviously improve olfactory function with different aspects, such as OT, ODI, and OI. The effect of DBS may be illustrated by the mechanism of indirect involvement of these cerebellar-efferent pathways after DBS. The olfactory dysfunction improvement with DBS is caused by the effects of DBS on the olfactory motor loop (19, 20). Within this circuit, the cerebellum is believed to maintain a feedback loop that regulates the sniff volume inversely proportional to odor concentration, allowing for optimal collection of olfactory information. The optimal efferent pathway after DBS may prevent patients from taking more vigorous sniffs at low n-butanol concentrations to detect the odor (21).

However, not all PD patients with hyposmia have satisfactory therapeutic effects, and thus some researchers consider DBS to have no effect on olfactory dysfunction (8, 22). After reviewing these papers, we considered the main contradictory results from the limited study samples (sample size <15) and observation time (post-operation time <3 months).

To explore the reason for the unsatisfactory improvement with DBS, our team found that PD with constipation has a greater risk of hyposmia and exhibits less improvement after DBS operation. These results cannot be explained well by the former model, which considered olfactory impairment to only originate from the central nervous system (CNS) but also peripheral nervous system (PNS). Therefore, the question was how hyposmia and constipation influence each other in PD. Here, we adopt the idea that patients with PD have highly heterogeneous symptoms and display capricious involvement of different neural regions, which can be partly explained by defining PD into a PNS-first and a CNS-first subtype (23). Based on this view, we defined PD patients with both olfactory dysfunction and constipation as the PNS-first subtype, in which the olfactory bulb and vagus nerve were attacked first and then α-syn retrograded along with the nerve to the central nervous system, resulting in the emergence of motor symptoms. A recent finding suggested that the risk of PD was significantly decreased in patients undergoing vagotomy (24). It appears that DBS may have a better therapeutic effect on hyposmia in the CNS-first subtype according to our theory, so we hope to demonstrate this in the next study.

Another question is regarding which factors lead to PNS-type PD. We considered that dysbiosis of the microbiota was vital for PNS-PD progression (25). Based on previous studies, human microbiota plays an important role in modulating the inflammatory milieu of the central nervous system in PD (26). Some environmental factors, such as caffeine consumption and cigarette smoking, may alter the composition of the gut microbiota in a way that influences intestinal inflammation (27). This, in turn, results in less aggregation of α-synuclein in the enteric nervous system (ENS), thereby lowering the risk of PD. This finding is in agreement with the observations of altered intestinal microbiota in PD patients and the profound influence of gut microbiota on the activity of ENS (28). To date, an increasing number of studies on NMS have focused on the popular “gut-brain axis” and confirmed the influence of microbiota. Our study also indicated that the difference in microbiota in nasal mucosa was different in PD patients with different NMS, and the compositions were determined by where the patients lived. This led to another interesting question whether there are some differences in the NMS between regions. If a PD patient lives far away from the sea, the question arises whether he will have a higher risk of developing hyposmia and constipation in the early stage of the disease.

Overall, the mechanism in which STN-DBS modulates brain circuitry and could influence the non-motor symptoms such olfaction is still not entirely understood. To our knowledge, the reason why constipation effected STN-DBS on hyposmia is that constipation usually indicated a dysbiosis of microbiota and this disorder usually impaired the olfactory bubble directly not the odor identification cortex in brain. STN-DBS mainly modulates circuitry and plastic change in brain with little therapeutic effect on peripheral nerve. The main strength of our study is that it is the first study to report the relationship between constipation and hyposmia in PD patients undergoing DBS surgery. However, the follow-up period of most studies was limited to 3 months; thus, only the acute effects of STN-DBS could be observed, which can be considered a weakness of the study. Besides, one paper (29, 30) indicated that the differentially abundant flora of the gut and oral microbiome in PD are more meaningful for NMS research; we will collect stool samples from PD patients to further explore the association between hyposmia and constipation in our next study.

Despite these limitations, our study suggests that DBS could improve hyposmia in PD patients, and the effect was influenced by whether the patient had constipation or not. This may be explained partially by the dysbiosis of microbiota in PD patients, which could also be helpful to call on the importance of early stage PD and present some hope to put off the hyposmia resulting from the neurodegeneration process.

## Conclusion

PD patients with constipation had a higher risk of hyposmia and experienced less therapeutic effect of STN-DBS on olfactory function improvement, which might be associated with dysbiosis of microbiota.

## Data Availability Statement

The raw data supporting the conclusions of this article will be made available by the authors, without undue reservation.

## Ethics Statement

The studies involving human participants were reviewed and approved by Qilu Hospitals' ethics committee approved study protocol (protocol number: KYLL-202008-065). The patients/participants provided their written informed consent to participate in this study. The animal study was reviewed and approved by Qilu Hospitals' ethics committee approved study protocol (protocol number: KYLL-202008-065).

## Author Contributions

YH, XW, Y-xL, and FL summarized the clinical cases and papers. CL wrote the main part of the manuscript including introduction results, methods and materials, and abstract. CZ and W-gL revised the paper. All authors reviewed the manuscript.

## Conflict of Interest

The authors declare that the research was conducted in the absence of any commercial or financial relationships that could be construed as a potential conflict of interest.

## Abbreviations

SST: Sniffin&#8217 Sticks odor test
PD: Parkinson's disease
STN: subthalamic nuclei oriented
OT: odor threshold
ODI: odor discrimination
OI: odor identification
TDI, threshold, discrimination: identification.
